# High-fidelity model to predict heat transfer enhancement for liquid film boiling on uniform micro-structured wicking surfaces

**DOI:** 10.1093/nsr/nwae148

**Published:** 2024-04-17

**Authors:** Vishwanath Ganesan, Mohammad Jalal Inanlu, Nenad Miljkovic

**Affiliations:** Department of Mechanical Science and Engineering, University of Illinois Urbana-Champaign, USA; Department of Mechanical Science and Engineering, University of Illinois Urbana-Champaign, USA; Department of Mechanical Science and Engineering, University of Illinois Urbana-Champaign, USA; Department of Electrical and Computer Engineering, University of Illinois Urbana-Champaign, USA; Materials Research Laboratory, University of Illinois Urbana-Champaign, USA; International Institute for Carbon Neutral Energy Research (WPI-I2CNER), Kyushu University, Japan; Institute for Sustainability, Energy and Environment (iSEE), University of Illinois Urbana-Champaign, USA

The field of thermal management in high-power electronic devices is a critical area of study, focusing on the efficient dissipation of heat to prevent device overheating and failure [1]. A key aspect of this field is understanding the dynamics of thin liquid film boiling, both on uniform and non-uniform micro-structured wicking surfaces, which can significantly enhance the heat transfer effectiveness [2]. This involves the study of complex thermal-hydrodynamic processes where vapor bubbles and capillary-driven liquid films interact to achieve a high heat transfer coefficient (HTC) and critical heat flux (CHF) on wicking structures [3]. The development and optimization of micro-structured surfaces [4], such as micropillars, micropowders and micromeshes, play a pivotal role in improving the thermal management of high-power devices [5]. Researchers in this field combine experimental insights with theoretical models to advance cooling technologies, contributing to the longevity and reliability of electronic devices [6,7].

**Figure 1.**
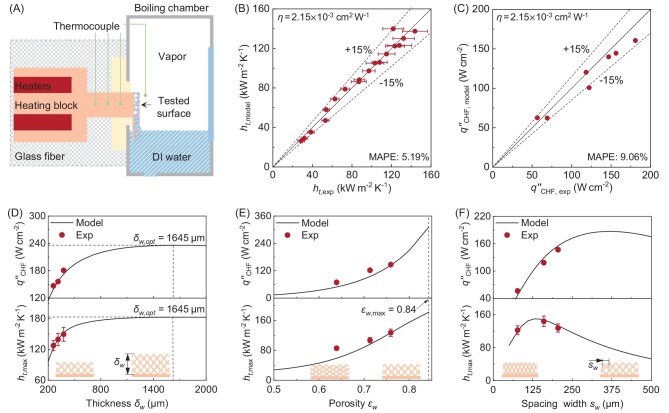
Determination of effective microlayer evaporation factor *η* by experimental results. (A) Schematic of the custom-made experimental set-up for liquid film boiling measurement. (B) Determination of *η* = 2.15$\ \times \ $10^−3^ cm^2^ W^−1^ with experimental data [8] on copper micromesh samples. (C) Comparison of experimental CHF and model-predicted CHF with *η* = 2.15$\ \times \ $10^−3^ cm^2^ W^−1^. The CHF and the maximum HTC as a function of (D) thickness ${{\delta }_w}$, (E) porosity ${{\varepsilon }_w}$ and (F) spacing width ${{s}_w}$. The red circles in (D–F) represent the experimental data and the black solid lines are the modeling results with *η* = 2.15$\ \times \ $10^−3^ cm^2^ W^−1^. Adapted from ref. [8] under the terms of the Creative Commons Attribution License (CC BY).

Recently, a team led by Professor Ronggui Yang at the School of Energy and Power Engineering, Huazhong University of Science and Technology, presented a high-fidelity thermal-hydrodynamic model in *National Science Review* [8] to simultaneously predict both HTC and CHF during liquid film boiling on uniform micro-structured wicking surfaces. The model accounts for the thermal-hydraulic and interfacial processes by considering both evaporation from the thin-film region atop the wick and nucleate boiling inside the wick. The universality of this model is witnessed through a scaling factor (*η*) independent of structural parameters (thickness ${{\delta }_w}$, porosity ${{\varepsilon }_w}$ and spacing width ${{s}_w}$) to characterize the change in microlayer evaporation with heat flux during nucleate boiling (Fig. 1). This scaling factor *η* = 2.15$\ \times \ $10^−3^ cm^2^ W^−1^ is empirically obtained by fitting the model predictions to their experimental results.

The researchers further delve into the complexities of developing general analytical expressions for both HTC and CHF during thin liquid film boiling on micro-structured wicking surfaces. The high-fidelity nature of these models is witnessed as they simplify and reduce to previously reported models under certain assumptions and limiting conditions. Using the unified scaling factor for microlayer evaporation, *η* = 2.15$\ \times \ $10^−3^ cm^2^ W^−1^, the model predictions of both HTC and CHF are in good agreement (well within mean absolute percentage error of ±30%) with the experimental data for various uniform micro-structured wicking surfaces reported in the literature. The wicking structures included silicon micropillar arrays, packed copper micropowders and sintered copper micromeshes. All three possible liquid supply methods of one-side, two-sides and all-around, were also included.

This research provides a very robust, high-fidelity and universal predictive tool for HTC and CHF during liquid film boiling on uniform micro-structured wicking surfaces. The tool can be extensively used for designing and optimizing structured surfaces for improved thermal management in electronic, energy and space systems. Additionally, the underlying theory used for modeling the nucleate boiling and thin-film evaporation heat transfer mechanisms can be further extended to predict heat transfer enhancement for both pool boiling [9] and flow boiling [10,11] on micro- and nano-structured surfaces. The integration of experimental insights and theoretical modeling in this study represents a significant contribution to the field of materials science and mechanical engineering, offering new avenues [12] for the development of advanced cooling technologies.
